# Clinical validation of using a commercial synthetic-computed tomography solution for brain MRI-only radiotherapy treatment planning

**DOI:** 10.1016/j.tipsro.2025.100328

**Published:** 2025-08-20

**Authors:** Lamyaa Aljaafari, Richard Speight, David L. Buckley, David Bird, Bashar Al-Qaisieh

**Affiliations:** a Leeds Institute of Cardiovascular & Metabolic Medicine (LICAMM), University of Leeds, Woodhouse, Leeds LS2 9JT, United Kingdom; b Department of Medical Physics and Engineering, Leeds Teaching Hospitals NHS Trust, Leeds LS9 7TF, United Kingdom; c King Saud bin Abdulaziz University for Health Sciences, Department of Diagnostic Radiology Faculty of Applied Medical Sciences, Alahssa, Saudi Arabia; d Leeds Institute of Medical Research (LIMR), Wellcome Trust Brenner Building, St. James’s University Hospital, Beckett Street, Leeds LS9 7TF, United Kingdom

**Keywords:** MRI-only planning, synthetic-CT model, MRI, Brain, Artificial intelligence

## Abstract

**Background and purpose:**

MRI-only radiotherapy treatment planning (RTP) relies on synthetic-CT (sCT) images for dose calculation. This study evaluates the clinical feasibility of using a commercial sCT solution in brain RTP, MRCAT Brain, focusing on dosimetric accuracy and patient setup verification.

**Method and materials:**

For dosimetric evaluation, 93 patients with brain cancer who were treated with volumetric modulated arc therapy (VMAT) using a CT/MRI fusion workflow were included. sCT images were generated using MRCAT Brain. The sCT images were rigidly co-registered to the CT images. The clinical plan produced on the CT was recalculated on the sCT. Dosimetric accuracy was assessed by comparing dose differences in dose volume histogram (DVH) statistics for the planning target volume (PTV) and organs at risk (OARs).

For patient setup verification, 70 patients were included, and total of 572 cone beam CT (CBCT) registrations were performed with sCT and CT as reference images. The sCT matching accuracy was validated by comparing the translational and rotational differences between sCT-CBCT and CT-CBCT registrations.

**Results:**

The PTV mean dose difference between CT and sCT were 0.3 %, 0.4 %, and 0.2 % for D50%, D2%, and D98%, respectively. The OAR mean dose differences were less than 0.3 % for all OARs. 4 of 93 patients (4.3 %) showed gross dosimetric errors of greater than ± 2 %. 3/4 were caused by sCT error. For positioning verification, all results were between ± 1 mm and ± 1°.

**Conclusion:**

This study demonstrates the clinical feasibility of the MRCAT solution for brain MRI-only RTP, with dosimetric differences being clinically acceptable, along with submillimetre and sub-degree accuracy in patient setup verification.

## Highlights

• Brain MRI-only radiotherapy treatment planning is clinically feasible.
• The commercial MRCAT solution demonstrated dosimetric accuracy, with mean PTV and OAR dose differences of less than 0.4 %.
• Patient positioning verification using Elekta XVI system was within ± 1 mm and ± 1° in all directions.
• Quality assurance guidelines are required for the safe implementation.

## Introduction

Brain radiotherapy treatment planning (RTP) relies on magnetic resonance imaging (MRI) for target delineation owing to its superior soft tissue contrast compared to computed tomography (CT) [[1], [2], [3]]. However, CT simulation remains essential for dose calculation and patient positioning during treatment delivery. The use of both imaging modalities requires co-registration of MRI and CT images, which can result in registration errors [4]. There is growing interest in implementing MRI-only RTP to eliminate uncertainties arising from co-registration and to simplify the workflow. An important benefit of MRI-only RTP is to eliminate the need for a CT scan. The patient only needs a single MRI scan for both delineation and treatment planning reducing time spent in the hospital.

MRI-only RTP requires an accurate synthetic-CT (sCT) image to provide electron density for dose calculation and patient positioning verification by registering to CBCT during treatment. With improvements in artificial intelligence (AI), researchers have increasingly focused on deep learning-based sCT models due to their enhanced performance [[5], [6], [7]]. Four commercial solutions using AI models are available as CE-marked and FDA-cleared software to directly produce brain sCT image from MRI data: MRCAT (Philips, Amsterdam, Netherlands), syngovia RT (Siemens Healthineers, Erlangen, Germany), MRI Planner (Spectronic Medical, Helsingborg, Sweden), and MR-Box (Therapanacea, Paris, France).

Clinical validation of these brain sCT products has been reported, and some have already been implemented clinically in specialist centres [[8], [9], [10], [11], [12], [13], [14]]. Three studies evaluated the MRCAT solution, Emin et al. [8] clinically implemented MRI-only in two cohorts, 30 patients for commissioning and 30 for validation, reporting mean dose differences within ± 0.7 % for the planning target volume (PTV) and ± 1.3 % organs at risk (OAR). However, this study did not include a quantitative assessment of patient positioning. Ranta et al. [10] reported a mean PTV difference of ≤ 0.6 % and normal brain tissue differences of ≤ 1.7 %, of 50 patients with positioning accuracy ranging from −2 to 2.5 mm (translation) and −1° to 0.5° (rotation) evaluated in only 20 patients using Varian Eclipse. Similarly, another study reported a mean PTV difference of −0.5 % and an OAR difference of ≤ 0.7 % in a cohort of 18 patients; however, positioning accuracy was not evaluated [11]. In addition, Lerner et al. [15] clinically implemented the MRI Planner solution in a cohort of 21 patients, demonstrating dose differences range within ± 1 % for the PTV and OAR, along with excellent CBCT positioning accuracy with within ± 1 mm in translation using the Varian Eclipse image registration tool. However, previous evaluation relied on the treatment planning system (TPS) rather than dedicated positioning software used on a linac during treatment.

Other studies using Syngovia reported mean differences of 0.2 % [13,14] for the PTV and ≤ 1.6 % [14] for the OAR. However, patient positioning was evaluated using 2D/2D kV images with the Brainlab Exactrac system, showing translational shifts of up to 5 mm and rotational errors of up to 4°. To the best of our knowledge, none of the previous studies have compared sCT and CT for CBCT-based patient positioning using a commercially available positioning software system. Therefore, this study aims for the first time in the literature to evaluate the MRCAT solution in brain radiotherapy across a large cohort of 94 patients to assess its dosimetric accuracy, and to verify patient positioning using the Elekta XVI system, following the exact clinical workflow including its registration features, similarity measures, and tolerances.

## Methods and materials

### Patient cohort and image acquisition

Local approval was granted to include data from 93 retrospective patients who received brain radiotherapy for palliative or radical treatment. The inclusion criteria required that patients had been treated with volumetric modulated arc therapy (VMAT) and underwent both CT and MRI simulations as part of their clinical pathway, with synthetic CT images generated during their MRI simulation following the acquisition of a source MRI scan. The exclusion criteria for MRCAT sCT generation, include bone disease and large tattoos in the head area. A thermoplastic head mask with three reference points, laser positioning markers and neck and knee support were used to immobilise patients during MRI and CT simulations, as well as throughout the radiotherapy treatment sessions. CT and MRI scans were conducted within a few hours to minimise positioning errors. The image acquisition detailed are in Table 1.

**Table 1:** shows the image acquisition details for CT, MRI, and CBCT.

Image acquisition	Parameters		Make & Model
CT	Resolution (mm^3^)	1.2 x 1.2 x 2	Philips Brilliance Big Bore (Koninklijke Philips N.V., the Netherlands)
	Tube voltage kVp	120	
	Tube current (mAs)	295	
	FOV	600 x600 x236	
	Scan time (s)	15.2	
MRI	Sequence	T1w 3D mDIXON FFE	1.5 T MR-RT scanner Philips Ingenia (Koninklijke Philips N.V., Amsterdam, the Netherlands)
	Acq. voxel size (mm^3^)	1.1x1.1x1.4	
	Recon. voxel size (mm^3^)	0.6x0.6x1	
	TE1/TE2 (ms)	2/4.4	
	TR (ms)		
	Flip angle (◦)	12	
	BW [Hz/px]	481	
	FOV (mm^3^)	232 × 270 × 260	
	Scan time (min: s)	2:56	
CBCT	Resolution (mm^2^)	1 x 1 mm	XVI software (Elekta, Stockholm, Sweden)
	Slice thickness	2 mm	
	kVp (kV)	120	
	Tube Current (mA)	0.4	
	Scan time (min)	1	

Abbreviation: computed tomography (CT), magnetic resonance imaging (MRI), acquisition (Acq), reconstruction (Recon), bandwidth (BW), pixel (px), echo time (TE), repetition time (TR), minutes: seconds (min:s), field of view (FOV) and cone beam CT (CBCT),

### sCT generation

The sCT images were generated using MRCAT brain, a commercially available product based on a deep learning algorithm (magnetic resonance for calculating attenuation, MRCAT Brain, version 4.0; Philips Oy, Vantaa, Finland). The algorithm uses a dedicated MRI acquisition, the 3D T1-weighted mDIXON XD FFE MRCAT source scan, which provides in-phase, fat-only, and water-only images to generate MRCAT image. The MRCAT algorithm is based on a convolutional neural network trained on matched pairs of CT and MRI data [16]. The sCT images automatically reconstructed from the MRCAT source MRI and provide relevant density information for dose calculation as part of the post-processing, with no user input.

### Plan generation

The CT, sCT, clinical treatment structures and plan were imported for each patient into RayStation Research version 12A-SP1 ML (RaySearch Laboratories, Stockholm, Sweden). The target was delineated on MRI, and the organs at risk (OAR) on CT. All patients were treated with VMAT plans with 4–5 mm PTV margin. The standard prescribed dose for radical treatment of brain tumours (e.g., glioma or meningioma) ranges from 50.4 Gy to 60 Gy delivered over 28–30 fractions; for palliative treatment, it ranges from 30 Gy to 40 Gy over 6–15 fractions. A beam energy of 6 MV flattening filter-free (FFF) and a dose grid of 3 × 3 × 3 mm^3^ were used per our clinical protocol for external beam treatment. The sCT images were rigidly co-registered with 6 degrees of freedom to the CT and resampled into the CT frame of reference to ensure accurate alignment of image intensities and internal anatomical positioning between the sCT and CT scans. The planning target volume (PTV) and OAR contouring were then transferred from the CT onto the sCT, except for the external contour, it can be automatically generated in RayStation. The clinical plan calculated on the CT without optimisation was then recalculated on the sCT for evaluation.

### Dosimetric evaluation

A dosimetric comparison between CT and sCT was performed using dose-volume histogram (DVH) statistics for the PTV and OARs. DVH statistics were selected according to our local clinical protocol and in line with ICRU Report 83 guidelines [17]. For the PTV, the analysis included D50, D2, and D98. For the OARs, the evaluated DVH statistics included: Dmean and D0.1 cc for the following OARs: brainstem, optic chiasm, optic nerves (left and right), orbits (left and right, and Dmean only for: cochlea (left and right), lacrimal glands (left and right), and lenses (left and right). The dosimetric differences between sCT and CT were computed using the following formula:

$$\Delta D\;=\;\frac{DsCT\;\;-\;DCT\;\;}{Dpres}\times 100\%$$

where ΔD is the dose difference on the DVH statistics, D_sCT_ and D_CT_ are the doses on sCT and CT, respectively, and D_pres_ is the prescribed dose.

### Patient set‐up verification

#### Reference data preparation

70 patients who received brain radiotherapy were included to assess the accuracy of patient positioning verification, using sCT compared to CT as the reference image on treatment delivery. 286 CBCTs were registered to the CT and sCT, respectively. For 47 patients, 5 CBCTs were evaluated; however, due to missing data, 5 patients had 4 CBCTs, 3 patients had 3 CBCTs, and 15 patients had only 1 or 2 CBCTs. A new treatment plan was generated on the sCT, using the same isocentre coordinates as the CT plan to ensure consistent reference positions for the matching. sCT, CT, and CBCT datasets were imported into the clinical XVI software. Image registration was performed using the clinical XVI software version 5.0 (Elekta, Stockholm, Sweden), replicating the clinical treatment process.

#### CBCT matching process

**Fig. 1:**
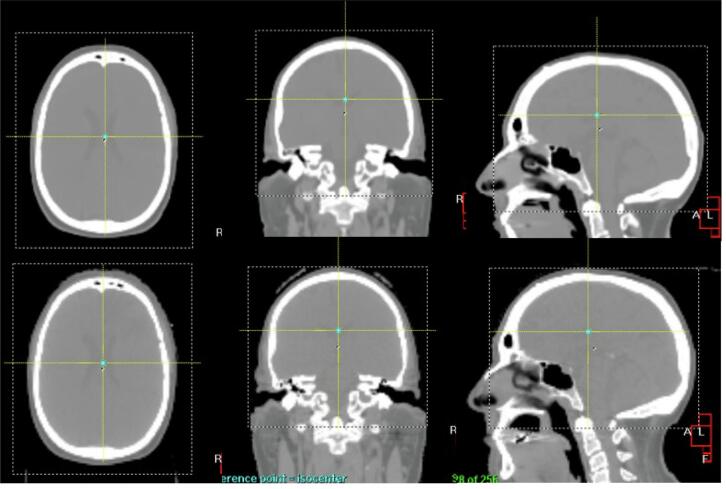
Example of image registration clipbox positioned on a reference, sCT (top row) and CT (bottom row) images for a brain patient in XVI (Elekta). The axial slice in (left), coronal (middle), and sagittal (right).

572 image registrations were re-performed offline on CBCT and CT, and CBCT and sCT, following the local matching protocol. A clip box was used to include the skull and skull base while excluding the C-spine. Fig. 1 shows an example of a brain clip-box used for a CT and sCT reference. An auto-matching grey value algorithm was applied for all patients using rigid registration. All registrations were performed by a single operator to ensure consistency across cases and eliminate inter-operator variability from the clinical workflow. The operator first registered all CT data, then repeated the registration on the sCT data with two days interval.

Positional errors were corrected in six degrees of freedom: three translations (measured in mm) and three rotations (measured in degrees). Translations were evaluated along the x, y, and z axes (left–right, anterior-posterior, superior-inferior), while rotations were assessed along the x (pitch), y (yaw), and z axes (roll). The mean difference was calculated for each axis. The systematic error in image registration between the two references, sCT and CT, were computed using the following formula:

$$\Delta T=\frac{TsCT}{CBCT}-TCT/CBCT\;\;$$

$$\Delta R=\frac{RsCT}{CBCT}-\;RCT/CBCT\;\;\;$$

where ΔT represents the translational difference in all directions (X, Y, Z), and ΔR represents the rotational difference in all directions (pitch, roll, yaw).

### Statistical analysis

Paired two‐tailed t‐tests were used to calculate 95 % confidence intervals on the mean sCT-CT differences for dosimetric accuracy (defined as dosimetric uncertainty of sCT being within ± 2 % of CT) and for patient positioning to assess systematic error between the two reference images.

## Results

### Dosimetric evaluation

**Fig. 2:**
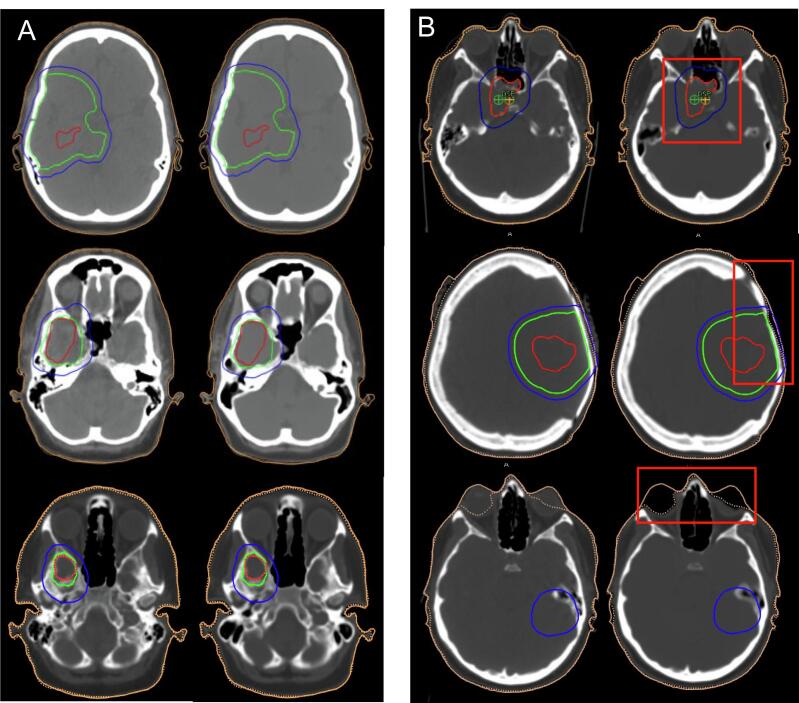
(A) Axial slices of CT (left) and sCT (right). Based on expert visual assessment, sCT images demonstrate reliable anatomical detail comparable to conventional CT scans. (B) Axial slices from three patients demonstrating dosimetric errors due to gross sCT anatomical failures, resulting in MRCAT images that are unacceptable for clinical use, CT (left) and sCT (right). The top and middle images show sCT errors in the PTV (bone), while the bottom image shows an sCT error in the OAR (orbits and lens). The structure contours are shown as PTV (blue) CTV (green), and GTV (red). (For interpretation of the references to colour in this figure legend, the reader is referred to the web version of this article.)

Fig. 2 shows example axial slices in CT and sCT for brain patients. Four patients showed significant dosimetric errors of ± 2.0 % or larger. When visually assessed 3 out of 4 were associated with sCT anatomical errors. The mean dose differences of the DVH statistics and the 95 % confidence interval range are shown in Table 2. The PTV mean dose difference were 0.3 %, 0.4 %, and 0.2 % for D50%, D2%, and D98%, respectively. Two patients demonstrating a PTV dose difference of ≥ ± 2.0 %, due to missing bone in the skull base or nasal bone, are shown in Fig. 2. The 95 % CI falls within a lower bound of 0.1 % and an upper bound of 0.5 %.

**Table 2:** PTV and OAR mean dose difference and the 95% confidence interval range between sCT and CT.

Structure (number of patients)	DVH statistics (Gy)	Mean dose difference (95 % CI lower, upper bound) (%)
PTV (n = 93)	D50%	0.3 (0.2, 0.3)
	D2%	0.4 (0.3, 0.5)
	D98%	0.2 (0.1, 0.2)
Brainstem (n = 93)	Dmean	0.2 (0.2, 0.2)
	D0.1 cc	0.3 (0.3, 0.4)
Optic Chiasm (n = 90)	Dmean	0.2 (0.2, 0.3)
	D0.1 cc	0.3 (0.2, 0.3)
Pituitary (n = 79)	Dmean	0.2 (0.2, 0.3)
Optic Nerve L (n = 93)	Dmean	0.2 (0.1, 0.2)
	D0.1 cc	0.2 (0.2, 0.3)
Optic Nerve R (n = 92)	Dmean	0.2 (0.1, 0.2)
	D0.1 cc	0.3 (0.2, 0.3)
Orbit L (n = 92)	Dmean	0.0 (0.0, 0.0)
	D0.1 cc	0.1 (0.1, 0.1)
Orbit R (n = 92)	Dmean	−0.1 (−0.1, 0.0)
	D0.1 cc	0.1 (0.0, 0.1)
Cochlea L (n = 93)	Dmean	0.2 (0.1, 0.3)
Cochlea R (n = 93)	Dmean	0.2 (0.1, 0.3)
Lacrimal Gland L (n = 93)	Dmean	−0.2 (−0.2, −0.1)
Lacrimal Gland R (n = 93)	Dmean	−0.1(−0.2, 0.0)
Lens L (n = 91)	Dmean	0.0 (−0.1, 0.0)
Lens R (n = 91)	Dmean	−0.1(−0.3, 0.1)

Abbreviation: planning target volume (PTV), mean dose (Dmean), Left (L), and Right (R), and confidence interval (CI).

**Fig. 3:**
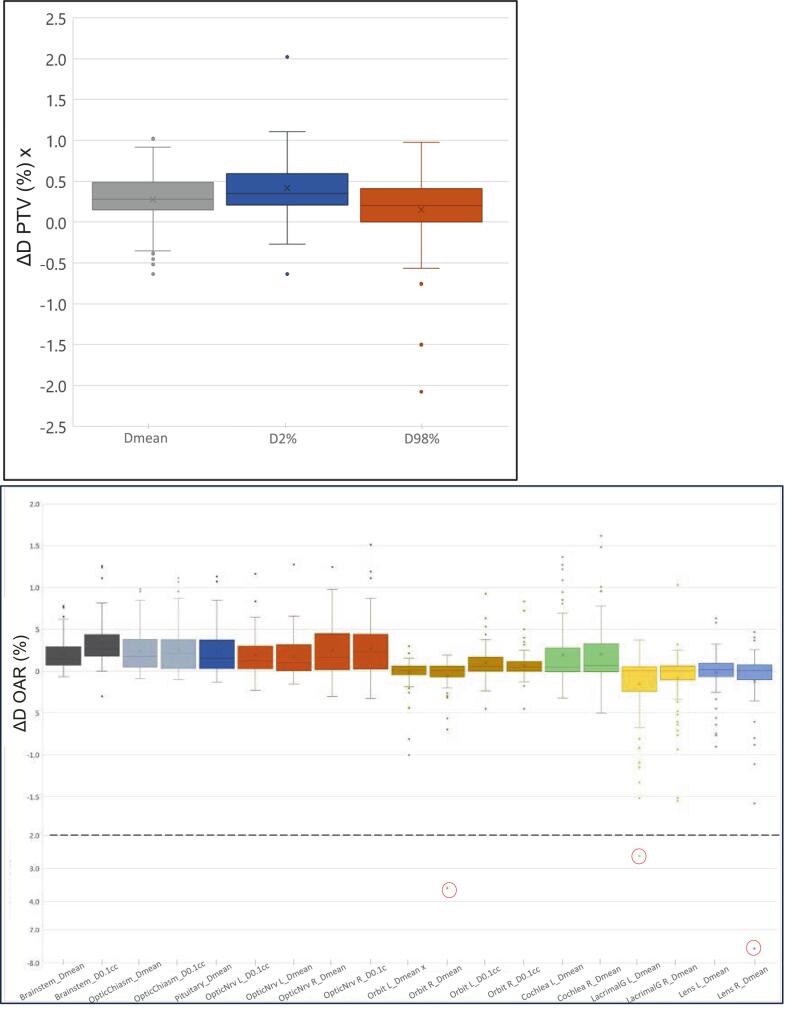
Boxplots for (A) PTV and (B) OARs show dose differences (%) between CT plans and sCT recalculations on the Y-axis. The X-axis represents the contour and specific DVH. The box represents the interquartile range (IQR), the horizontal line indicates the median, whiskers show values within 1.5 times the IQR, and points are outliers. The red circles show the larger outliers. Abbreviation: planning target volume (PTV), organ at risk (OAR)mean dose (Dmean), Left, R (L), Right (R) optic nerve (OpticNrv), and Lacrimal gland (LacrimalG). (For interpretation of the references to colour in this figure legend, the reader is referred to the web version of this article.)

The OAR mean dose differences were less than 0.3 % for all OARs. Notably, large outliers were observed in two patients who had dose differences greater than ± 2.0 %. One patient showed dose differences of −3.6 % and −7.6 % in the right orbit and lens, respectively due to signal void in MRI caused a sCT error. However, the second patient showed a dose difference of −2.6 % in the left lacrimal gland, which is considered less significant due to its small volume being located on a dose gradient and was not caused by sCT anatomical errors. The 95 % CI falls within a lower bound of −0.3 % and an upper bound of 0.4 %. Fig. 3 shows the distribution of dose differences (%) between sCT minus CT in DVH statistics for PTV and OAR.

### Patient set‐up verification

**Fig. 4:**
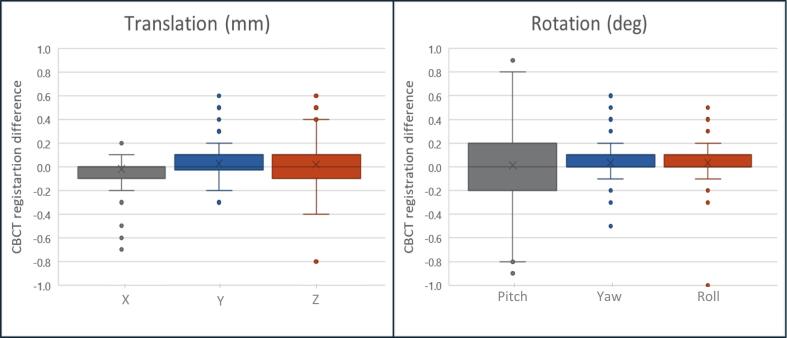
Shows box plots position error differences between cbct-sct minus cbct-ct for (a) translation and (b) rotation in all direction. the box represents the iqr, the horizontal line indicates the median, whiskers show values within 1.5 times the iqr, and the outliers represented as points.

Fig. 4 shows the box plots of positional differences between CBCT-CT and CBCT-sCT in translation and rotation. The mean positional differences in all directions were 0.0 mm for translation and 0.0° for rotation. The range of translational shifts were between [0.2: −0.7 mm], [0.6: −0.3 mm], and [0.6: −0.8 mm] in the X, Y, and Z directions, respectively. The range of rotational shifts were between [0.9: −0.9°], [0.6: −0.5°], and [0.5: −1°] in the pitch, yaw and roll, respectively. The 95 % confidence intervals were ranging from 0.04 mm to −0.04 mm and 0.03° to −0.05° for translations and rotations, respectively.

## Discussion

This study aims to assess the dosimetric accuracy and patient setup verification of MRCAT Brain and demonstrate its clinical feasibility for MRI-only RTP. Compared to previous studies, this study evaluated a large cohort of 94 patients and demonstrated the model's generalisability and suitability for brain MRI-only RTP. In addition, the quantitative assessment of patient setup verification in CBCT registration using a commercially available positioning software system remains unaddressed. This study, for the first time in the literature, evaluated the performance of the Elekta XVI system on a cohort of 70 patients to ensure it is suitable for using sCT as a reference image. Overall, dosimetric and patient setup evaluations confirm that MRCAT produces accurate sCT images, making it a reliable solution for brain MRI-only radiotherapy. Brain MRCAT met all evaluation criteria, with no clinically significant difference indicated by 95 % confidence intervals (considered to be <±2%) [18].

Multiple commercially available solutions have been introduced by vendors for clinical use. Several studies have assessed their clinical feasibility and implementation, reporting mean dose differences ranging from −0.7 % to 0.5 % for PTV and −1.4 % to 1.7 % for OARs [8,10,[12], [13], [14], [15],^19^]. Compared to these findings, this study found significantly better mean dose differences in the PTV and all relevant OARs.

Although mean dose differences were found to be clinically acceptable, there were two notable outliers in PTV dose differences (2 % for D_2_% and −2.1 % for D_98_%). These two cases are not suitable for clinical use due to gross sCT failures, mainly due to missing bone at nasal cavity or surgical sites. In Case 1, there was no clear cause visible on the MRI; however, this region is known to be challenging due to the presence of both air and bone, which may have contributed to inaccurate bone reconstruction by the MRCAT model. A key reason is that MRCAT relies on Dixon MRI sequences, which differentiate between fat and water but do not provide direct bone contrast, especially for small bones and dense tissue. In Case 2, the patient had undergone major surgery, and a section of the skull was replaced with a metal mesh. Although the MRI has no obvious artefact, the anatomy was likely very different from usual bone structure, which may have caused MRCAT to fail in reconstructing the bone correctly in that region. Therefore, to clinically implement MRCAT Brain, an effective quality assurance (QA) method is needed to identify patients who may experience these dose differences.

OARs outliers were also observed in two patients, with dose differences exceeding ± 2 %. The first patient showed a dose difference of −3.6 % in the right orbit and −7.6 % in the right lens. This patient's MRCAT source showed a signal void, possibly due to eye cosmetics distorting the magnetic field, which led to the complete loss of soft tissue in the right orbit and partial loss in the left orbit. This resulted in a significant anatomical sCT failure and led to dosimetric errors in the eye regions. The second patient showed a dose difference of −2.6 % in the left lacrimal gland due to the gland’s small size being located on a steep dose gradient next to bone with slight difference in bone densities. In addition, there was patient positional difference, resulting in external body contouring discrepancy of 0.6 cm on average, contributing to the observed dose difference. Our results showed smaller mean dose differences compared to previous studies that included these OARs. One study reported mean dose differences of ≤ ±0.7 % in orbit, lens, and cochlea [19]. Another study found −1.1 % and −0.9 % in the left and right cochlea, respectively [8]. These regions are among the most challenging in MRI-only brain radiotherapy due to their small size, sensitivity to positional differences, and bone and air interfaces. As shown in Fig. 3, the sCT plans delivered colder dose in the lacrimal gland and cochlea of up to −1.5 % compared to CT plans, indicating systematic difference in the sCT plans, as these are bone heavy regions where it is challenging to obtain accurate density information. Although our results show that these differences are considered clinically acceptable, it is important to note that they are more uncertain than those observed in other OARs and PTVs.

In general, common artifacts associated with sCT generated from AI were observed in the brain [9,[20], [21], [22]]. These artifacts include issues such as missing nasal bone, and skull bone due to surgical clips; differences in the external contour due to patient positioning; variations in bone density in the ear canal; and misclassification of bone and air. However, these artifacts were small and clinically insignificant, considering dose differences between sCT and CT within ± 2 %.

For patient setup verification, the translational and rotational differences between sCT/CBCT and CT/CBCT were less than ± 1 mm and ± 1°in all directions. These results are comparable to, or better than, reported studies based on CBCT patient positioning using Eclipse TPS which range from −0.3 to 0.5 mm [12,15] and −2 to 2.5 mm [10], and −0.3 to 0.3° [12] and −1 to 0.5°[10]. In contrast, two studies based on 2D/2D kV imaging using Brainlab observed larger differences, with translational shifts of 5 mm and rotational errors of 4°[13,14]. In this study, the potential errors in the systematic differences were very small, as indicated by the narrow 95 % confidence intervals.

When correlating the dosimetric outliers with patient setup accuracy, no significant impact was found. Since the brain is a rigid organ, and image registration primarily relies on bony structures. Minor artifacts such as a missing frontal bone or anatomic anomalies due to surgery did not compromise the overall quality of CBCT- registration. Although the residual errors were small, we observed that the outliers in the X-direction came from a single patient across multiple fractions, similarly in the Y and Z directions. Therefore, the sCT images generated from MRCAT are an accurate alternative to CT reference for CBCT image registration during treatment, it exceeds acceptable standards with sub-millimetre accuracy.

Despite the overall accuracy was clinically acceptable, four cases (representing 4.3 % of cases) showed significant dosimetric errors in the sCT, were 3 out 4 cases showed sCT anatomical errors, making them unsuitable for clinical use. Therefore, this study confirms the necessity to establish a comprehensive QA program to ensure safe clinical implementation for MRI only RTP [20]. Patient-specific QA should be performed by radiographers after MRI simulation to detect any significant sCT artifacts. A second check should then be conducted by medical physicists or dosimetrists prior to treatment planning. It is important to note that AI-generated sCT models may introduce errors and are not always completely reliable. For example, as shown in Fig. 2(B) middle images, post-surgical changes may cause a minor artifact, such as thinner bone in the skull region, that might be missed during visual assessment alone. Therefore, additional verification steps, such as recalculating dose distributions using CBCT, are recommended to ensure the reliability and accuracy of sCT-based treatment plans. A local QA protocol for sCT commercial solutions has been introduced in the literature [8,9,15], highlighting visual assessment of any artifacts that may compromise the image quality of sCT or treatment planning as discussed above.

## Conclusion

This study demonstrated the clinical feasibility of brain MR-only treatment planning using the MRCAT solution as part of the commissioning process. In a large cohort of patients, all evaluated criteria were clinically acceptable with dose differences remained within ± 2 %, and patient setup verification achieved sub-millimetre and sub-degree precision in patient setup verification using a clinical CBCT registration software for the first time in the literature. However, in 3.2 % of cases, MRCAT failed to generate acceptable sCT, requiring careful checking before clinical implementation.

## Funding

This work was supported by the National Institute for Health and Care Research (NIHR), [NIHR Senior Clinical and Practitioner Research Award R1, NIHR304555] .

## Declaration of competing interest

The authors declare that they have no known competing financial interests or personal relationships that could have appeared to influence the work reported in this paper.
